# Isolated reefs support stable fish communities with high abundances of regionally fished species

**DOI:** 10.1002/ece3.7370

**Published:** 2021-03-16

**Authors:** Matthew J. Birt, Katherine Cure, Shaun Wilson, Stephen J. Newman, Euan S. Harvey, Mark Meekan, Conrad Speed, Andrew Heyward, Jordan Goetze, James Gilmour

**Affiliations:** ^1^ The Australian Institute of Marine Science Indian Ocean Marine Research Centre, Cnr of Fairway and Service Road 4 Perth WA 6009 Australia; ^2^ Marine Science Program Department of Biodiversity, Conservation and Attractions Government of Western Australia 17 Dick Perry Ave Kensington WA 6151 Australia; ^3^ Oceans Institute The University of Western Australia Indian Ocean Marine Research Centre, Cnr of Fairway and Service Road 4 Perth WA 6009 Australia; ^4^ Western Australian Fisheries and Marine Research Laboratories Department of Primary Industries and Regional Development Government of Western Australia P.O Box 20 North Beach WA 6920 Australia; ^5^ School of Molecular and Life Sciences Curtin University Perth WA Australia

**Keywords:** baselines, endangered fish, fish assemblages, remote reefs, stereo‐BRUVS, temporal stability

## Abstract

Anthropogenic impacts at isolated and inaccessible reefs are often minimal, offering rare opportunities to observe fish assemblages in a relatively undisturbed state. The remote Rowley Shoals are regarded as one of the healthiest reef systems in the Indian Ocean with demonstrated resilience to natural disturbance, no permanent human population nearby, low visitation rates, and large protected areas where fishing prohibitions are enforced. We used baited remote underwater video systems (BRUVS) to quantify fish assemblages and the relative abundance of regionally fished species within the lagoon, on the slope and in the mesophotic habitat at the Rowley Shoals at three times spanning 14 years and compared abundances of regionally fished species and the length distributions of predatory species to other isolated reefs in the northeast Indian Ocean. Fish assemblage composition and the relative abundance of regionally fished species were remarkably stable through time. We recorded high abundances of regionally fished species relative to other isolated reefs, including globally threatened humphead Maori wrasse (*Cheilinus undulatu*s) and bumphead parrotfish (*Bolbometopon muricatum*). Length distributions of fish differed among habitats at the Rowley Shoals, suggesting differences in ontogenetic shifts among species. The Cocos (Keeling) Islands typically had larger‐bodied predatory species than at the Rowley Shoals. Differences in geomorphology, lagoonal habitats, and fishing history likely contribute to the differences among remote reefs. Rowley Shoals is a rare example of a reef system demonstrating ecological stability in reef fish assemblages during a time of unprecedented degradation of coral reefs.

## Graphical abstract

Fish assemblage composition and the relative abundance of regionally fished species were remarkably stable through time at the Rowley Shoals. We recorded high abundances of regionally fished species relative to other isolated reefs, including globally threatened humphead Maori wrasse (*Cheilinus undulatus*) and bumphead parrotfish (*Bolbometopon muricatum*).

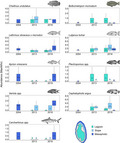

## 1 INTRODUCTION

Coral reef fisheries provide sustenance and livelihoods for millions of people (Pauly et al., 2005), yet uncontrolled fishing pressure on many of the world's reefs is threatening the stability of these fisheries (MacNeil et al., 2015; Newton et al., 2007). Managing reef resilience and multispecies coral reef fisheries are often premised on an understanding of fish assemblages in the absence of other anthropogenic pressures, particularly fishing. Remote reefs, with minimal human interaction and low historical fishing pressure, offer an ecologically meaningful baseline to compare with regions facing more severe human disturbances (Knowlton & Jackson, 2008).

The low fishing activity often associated with remote coral reefs can result in fish biomass higher than accessible reefs, even those with long‐standing and well‐enforced no‐take areas (McClanahan et al., 2019). High biomass estimates are often associated with a high abundance of larger‐bodied predatory fish (Quimbayo et al., 2017; Stevenson et al., 2007) that are typically targeted by fishers (Pauly et al., 1998). The ecological benefits of high fish biomass and abundant predators may flow through the system, improving overall ecosystem health and resilience (Friedlander & DeMartini, 2002; Jackson et al., 2001; Link & Watson, 2019). The high biomass often found at isolated coral reefs is also typically associated with high functional diversity of fishes, which maintains vital ecosystem processes (Mora et al., 2011). This includes rare species, whose ecological roles can be important despite their low abundance (Graham & McClanahan, 2013; Mouillot et al., 2013). However, remoteness may not always result in diverse and abundant fish assemblages and it is important to identify variation within and among isolated reefs (McClanahan et al., 2019). The combination of isolation and protection from fishing is rare; a large proportion of remote oceanic atolls lack fisheries protection, even when they are hot spots for fish and shark populations (Cinner et al., 2016; Letessier et al., 2019).

Remoteness, while an advantage in terms of impacts from human disturbances, can also increase vulnerability. Fish recruitment at isolated reefs is often dependent on locally produced larvae (Green et al., 2015; Underwood et al., 2012), with large‐scale oceanic dispersal generally reduced by geographical and physical barriers associated with large stretches of deep oceanic water (Luiz et al., 2012). Population persistence therefore depends on maintaining levels of spawning stock biomass, as external sources of recruitment are limited. Low connectivity among oceanic atolls can also make isolated fish assemblages more susceptible to inbreeding, which promotes low genetic diversity, reduces the capacity of local populations to respond and adapt to change (Almany et al., 2009; Frankham, 1996), and may increase local extinction risk (Dulvy et al., 2003). In a rapidly changing world, isolated reefs may therefore be vulnerable to the combined effects of warming waters, coral bleaching, altered oceanographic patterns, and increased storm frequency (Hughes et al., 2018; Puotinen et al., 2020).

Reefs naturally differ among habitats and depth gradients, due to differences in physical and oceanographic factors such as temperature, water movement, primary productivity, light availability, and physical orientation (Hamner et al., 2007; Ke et al., 2018; Moore & Morrison, 2009). These environmental factors shape the composition of benthic biota and associated fish assemblages. For example, reef predators often show a preference for outer reef slopes (Dale et al., 2011; Friedlander et al., 2010), where planktivorous fish prey are abundant due to enhanced primary productivity driven by oceanic currents (Skinner et al., 2019). On deeper reefs (>30 m), fish assemblages are also often ecologically distinct, with low abundance of herbivores, a concentration of predatory fish biomass, and high abundance of planktivorous fishes (Rocha et al., 2018; Stefanoudis et al., 2019). Furthermore, although pressures such as pollution may extend throughout a coral reef (Rocha et al., 2018), the impact may vary among habitats. The impacts from heat stress and damaging waves are typically greatest on shallow reefs (Frade et al., 2018). Deeper reefs (>30 m) are thought to provide areas where coral reef taxa can survive during periods of adverse conditions elsewhere (Bongaerts et al., 2015; Smith et al., 2014), and may assist recovery of shallow‐water reefs by restocking them with larvae (Bongaerts et al., 2015; Costantini et al., 2011; Vaz et al., 2016). However, mesophotic reefs are not immune to disturbance, and if they are to act as depth refuges, they need to be resilient and sustain populations that connect with those in other reef habitats (Abesamis et al., 2018; Bongaerts & Smith, 2019; Pinheiro et al., 2019). Understanding variability across reef habitats in protected isolated reefs subject to low levels of environmental disturbance may provide further insights into the overall capacity of reefs to survive rapid ongoing change under ecologically optimal conditions (Knowlton & Jackson, 2008; Skinner et al., 2020).

The Rowley Shoals is a cluster of three oceanic atolls located ~260 km from the Australian mainland that has no permanent human population, low visitation rates, and large no‐take marine reserves with regular compliance activities (MPRA, 2015). The Rowley Shoals' distance from the Australian coast and Indonesia has likely contributed to minimal historical fishing pressure in comparison with reefs further north (e.g., Ashmore Reef and Scott Reef; Edgar et al., 2017; Russell & Vail, 1988; Serventy, 1952). As a result, they are often referred to as “pristine” and are regarded as one of the healthiest reef systems in the east Indian Ocean (Allen, 2000; Field et al., 2011). The impacts of warming oceans and extreme oceanographic events such as El Niño on coral health in the Rowley Shoals are low compared with other reefs (Hughes et al., 2018). The reefs have undergone multiple cycles of impact and recovery from localized exposure to cyclones and damaging wave action, with an overall increase of ~30% in mean coral cover over the past 22 years (Gilmour et al., 2019). Despite their pristine status, studies assessing the stability of fish communities through time are limited (Ruppert et al., 2013), and none have investigated spatial differences among reef habitats and depths. Further, we know little of how abundances of species vulnerable to fishing compare among remote reefs in the region (but see Barley et al., 2017; Bennett et al., 2018; Edgar et al., 2017).

The aim of this study was to assess fish assemblages and regionally fished species across multiple reef habitats at the Rowley Shoals as a baseline for eastern Indian Ocean coral reef atolls. We also assess potential changes in fish assemblages and regionally fished species through time and compare these regionally fished species with other research campaigns that used the same methodology to sample remote oceanic coral reefs in the region with different geomorphology and fishing pressures. We use baited remote underwater video systems (BRUVS) to survey fishes, because they are not limited by depth, provide accurate estimates of length, and capture a wide diversity of reef fishes, particularly large predators often underrepresented in diver‐based visual surveys (Harvey et al., 2001; Watson et al., 2010). The objectives of this study are to (a) assess how fish assemblages at the Rowley Shoals differ among reef habitats and depths through time; (b) determine whether the assemblage and abundance of regionally fished species changed through time; (c) compare the abundance of regionally fished species at the Rowley Shoals to other remote reefs; and (d) compare the size of predatory species at the Rowley Shoals to other remote reef systems.

## 2 METHODS

### 2.1 Study site

**FIGURE 1:**
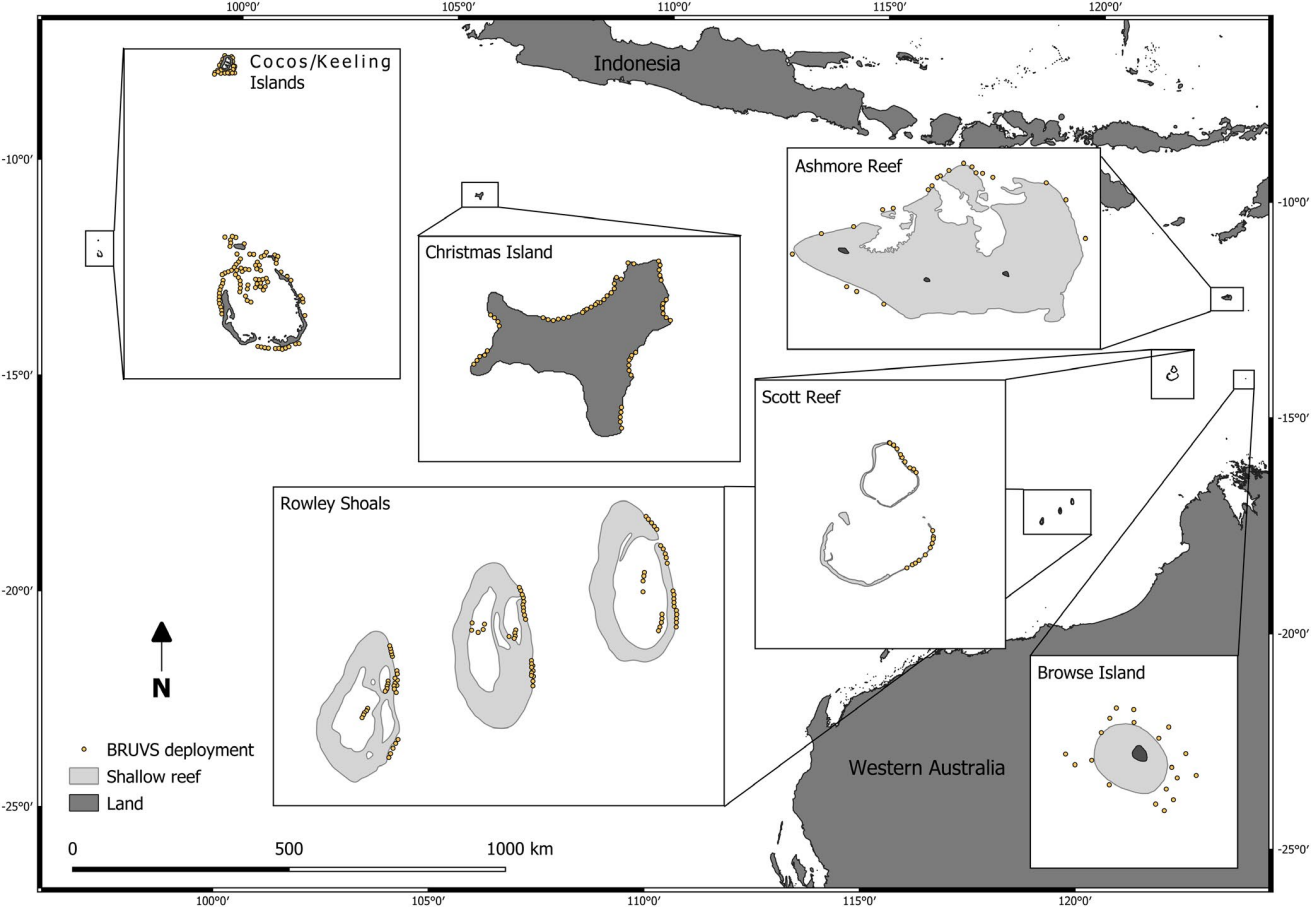
Map of all locations sampled and position of each BRUVS deployment. Note: Rowley Shoals are not to scale in inset map

The Rowley Shoals are located approximately 260 km offshore from mainland Western Australia between 17°07′S, 119°36′E and 17°35′S, 118°56′E (Figure 1). They consist of three uninhabited oceanic atolls (Imperieuse, Clerke, and Mermaid reefs) which are between 30 and 40 km apart. Clerke and Imperieuse are partially protected from fishing (24% of the marine park is zoned no‐take), and Mermaid is fully protected, totaling 752 km^2^ of no‐take zones across the three atolls (Table 1; MPRA & DEC, 2007). Although recreational and charter fishing is permitted in some areas, most (>80%) fish caught by charter operators are released and retained species are primarily pelagic (e.g., *Gymnosarda unicolor*, *Caranx melampygus*, *Thunnus albacares,* and *Acanthocybium solandri*; MPRA, 2015). All Epinephelidae (cod/groupers) and Labridae (wrasse) species are totally protected throughout the Rowley Shoals. Each atoll is similar in size, shape, and orientation, with outer reef flat and crest enclosing a lagoon (Collins, 2011).

**TABLE 1:** Characteristics of locations of BRUVS deployments (total 392) at six reefs, 2004–2018

Location	Year sampled	Lagoon BRUVS (# sites)	Slope BRUVS (# sites)	Deep BRUVS (# sites)	Camera type	System configuration	Island type	Local population	No‐take zones	Year protected	Enforcement
Rowley Shoals	2018	29(6)	29(6)	30(6)	GoPro Hero5	Stereo	Atoll	0	Yes	1990	Yes
	2013	25(5)	30(6)	—	Sony HDR‐CX110E	Mono					
	2004	—	—	20(4)	Sony DCR‐TRV18	Stereo					
Scott Reef	2016	—	20(2)	—	GoPro Hero4	Mono	Atoll	0	No	—	—
Browse Island	2018	—	12(1)	8(1)	GoPro Hero4	Mono/Stereo	Emergent limestone	0	No	—	—
Ashmore Reef	2016	—	21(3)	—	GoPro Hero4	Mono	Atoll	0	Yes	1988	Yes
Cocos (Keeling) Islands	2016/2018	33(3)	80(4)	—	GoPro Hero3+	Stereo	Atoll	544	Yes	1995	Yes
Christmas Island	2016	—	55(10)	—	GoPro Hero3+	Stereo	Emergent limestone	1,843	No	—	—

### 2.2 Comparison sites

The relative abundance of regionally fished species at the Rowley Shoals was compared with five other remote reefs in the eastern Indian Ocean, which had been sampled using standardized BRUVS methodology (Langlois et al., 2020): Scott Reef, Browse Island, Ashmore Reef, Cocos (Keeling) Islands, and Christmas Island.

Scott Reef (14°0′S, 121°45′E) comprises two oceanic atolls (Scott Reef North and Scott Reef South). It has been fished by Indonesian artisanal fishers since at least the 1800s and is still open to fishing under a memorandum of understanding (MOU) between the Australian and Indonesian governments (Meekan et al., 2006; Russell & Vail, 1988; Serventy, 1952). North Scott Reef has an enclosed lagoon surrounded by an outer reef flat and crest. South Scott is a horseshoe‐shaped atoll open to the ocean via a series of deep channels to the north, resulting in a deeper lagoon than at North Scott.

Browse Island (14°S7′S 123°33′E) has a land area of 0.17 km^2^ surrounded by a largely intertidal fringing reef platform up to 1 km wide, with no lagoons (Figure 1). Indonesian artisanal fishers are permitted to fish under the MOU agreement.

Ashmore Reef (12°15′S, 123°3′E) is a large lagoonal platform reef with three small uninhabited islands and two lagoons separated by a calcareous rise (Collins, 2011; Figure 1). Ashmore Reef was proclaimed a national reserve in 1983, although Indonesian artisanal fishing was permitted under the MOU until 1988. After 1988, fishing was banned except in the west lagoon where subsistence fishing is still permitted. However, illegal fishing likely occurred until a compliance vessel was deployed at Ashmore on a near‐permanent basis in 2008 (Table 1; Commonwealth of Australia, 2002; Field et al., 2009; Speed et al., 2018).

The Cocos (Keeling) Islands (12°12′S, 96°54′E) are two oceanic atolls with 27 islands. The southern Cocos (Keeling) atoll has a shallow lagoon with two northern passages connecting it to the open ocean (Figure 1). Cocos (Keeling) Island has a local population of approximately 544 people (Table 1; Australian Bureau of Statistics, 2016a, 2016b) and is open to fishing (Evans et al., 2016). The smaller unpopulated North Keeling Island 24 km to the north is a no‐take sanctuary covering approximately 25 km^2^ and has no lagoon (Director of National Parks, 2015; Hobbs & Newman, 2016).

Christmas Island (10°27′S, 105°41′E), an uplifted limestone cap metamorphized from coral reefs, has a local population of 1843 and is open to fishing (Table 1; Australian Bureau of Statistics, 2016b; Director of National Parks, 2014). Christmas Island has a narrow shelf and poorly developed fringing reefs with no lagoonal system (Bennett et al., 2018).

### 2.3 Sampling technique and design

We assessed variation in fish assemblages across reef habitats and depth with a comprehensive survey of the Rowley Shoals in 2018. We placed 4–5 replicate BRUVS within the lagoon (5–12 m depth), on the fore‐reef slope (5–14 m) and at mesophotic depths (40–75 m) at two sites at each of the three atolls (Figure 1). The BRUVS setup consisted of two GoPro Hero5 cameras (settings: 30 frames per second, 1,920 m × 1,080 pixel resolution, medium field of view) mounted 650 mm apart and inwardly converged at an angle of 5 degrees to allow for stereo measurements. Cameras were mounted in custom housings designed to maximize calibration stability. Each BRUVS was baited with 1 kg of crushed pilchards (*Sardinops* spp.) suspended in a plastic‐coated wire mesh bag 1.2 m in front of the cameras. Each deployment was separated by at least 250 m to minimize overlap of bait plumes and reduce the likelihood of fish swimming between samples (Cappo et al., 2001).

We sampled during daylight, at least one hour outside of crepuscular periods to minimize potential variability in fish assemblages with time of day (Birt et al., 2012; Myers et al., 2016). Each deployment was randomized in time with respect to the treatment and left to record for 60 min. The exception is the Browse Island slope samples, which were only deployed during the morning and should therefore be interpreted with caution as this may have influenced comparisons with other locations in the afternoon (Birt et al., 2012). We discarded two deployments due to limited field of view (facing substrate) resulting in a total of 88 deployments across the three atolls.

Historical surveys conducted at the Rowley Shoals that used the same BRUV methodology were used for temporal comparisons. In 2013, sampling was completed at the same two sites within the lagoon and fore‐reef slope habitats, except at Mermaid atoll, where only one site was sampled within the lagoon. There were no deployments in mesophotic habitats in 2013. In 2004, BRUVS were only deployed in the mesophotic habitat, sampling two sites at each of Clerke and Imperieuse atolls. Replicates were haphazardly placed in all years and habitats, except for lagoon sites in 2013 and 2018 where they were deployed using the same GPS coordinates. Therefore, statistical analyses in the lagoon followed a repeated‐measures approach.

To compare the Rowley Shoals to other isolated atolls, data were sourced from a range of different sampling programs, and therefore, the numbers of replicates per site and reef habitats surveyed differed (Table 1). However, the slope was sampled at all locations, the mesophotic habitat was sampled at Browse Island, and the lagoon was sampled at Cocos (Keeling) Island. Reef habitats were in similar depths in each location. Differences in design were accounted for in the statistical analyses (see Section 2.5). Logistical constraints meant that both the Rowley Shoals and Scott reef could only be sampled on the sheltered (eastern) sides of the atolls. Previous research (Raedemaecker et al., 2010; Floeter et al., 2007; Wilson et al., 2003) and our own preliminary comparison between exposed and sheltered sites at Cocos Island (Appendix S2) have revealed that although fish communities on sheltered and wave‐exposed reefs may differ, the regionally fished species examined here are likely at similar or higher abundances at exposed locations. Therefore, we expect observations on the sheltered sides of the Rowley Shoals and Scott Reef to yield conservative estimates of regionally fished species.

### 2.4 Image analysis

We used the EventMeasure software (www.seagis.com.au) to identify and count fish. To avoid repeated counts of the same fish and for standardization among historical and interinstitutional datasets, we counted the maximum number of individuals in the field of view at one time (MaxN) to estimate relative abundance (Cappo et al., 2003). At the Rowley Shoals, all fish were identified to species when possible. We compared species lists to minimize interobserver biases across the 3 years. Mismatches were checked against species lists from the Rowley Shoals maintained by the Western Australian Museum. Species that had not been confirmed were checked on videos and corrected if necessary or lumped to genus whether identification was uncertain. Data from 2004 were missing many small‐bodied species, likely unidentifiable due to the lower definition imagery available at the time (Sony DCR‐TRV18); therefore, we did not compare fish assemblages between the 2004 and 2018 datasets. Instead, we considered only selected regionally fished species in 2004 data. We identified *Variola* spp., *Ctenochaetus* spp., *Macolor* spp., and *Lethrinus olivaceus* × *microdon* as species complexes due to difficulties distinguishing between species within these genera from the available video imagery.

BRUVS were calibrated before deployment using the CAL software (www.seagis.com.au) following procedures outlined by Harvey and Shortis (1998). EventMeasure was then used to measure the fork length of selected predator species at MaxN, when fish were up to 10 m from the camera to standardize samples. Predator species selected included *Cheilinus undulatus* and those in the genera *Lethrinus, Scomberoides, Monotaxis, Epinephelus, Plectropomus, Lutjanus, Triaenodon, Variola, Caranx, Carcharhinus, Gymnocranius, Aprion, Cephalopholis, Macolor, Aphareus, Aethaloperca, Anyperodon, Galeocerdo, Symphorichthys, Gymnosarda, Symphorus, Sphyrna, Seriola, Elagatis, Negaprion, Sphyraena,* and *Gracila*. Comparable measurements were obtained from the Cocos (Keeling) Islands (the only other location with length data available) and used to assess differences in length distributions between locations.

### 2.5 Statistical analyses

We used multivariate permutational analysis of variance (PERMANOVA) to test for differences in the overall fish assemblage due to protection status (fished vs. unfished), year (2013, 2018), shoal (Imperieuse, Clerke, or Mermaid), and reef habitat (lagoon, slope, or mesophotic). Three designs were used in the multivariate PERMANOVA tests: (a) a 4‐factor design incorporating protection status (two levels; fixed), shoal (three levels; fixed), reef habitat (three levels; fixed), and site (random, nested in status, shoal, and reef habitat) to compare assemblages in different reef habitats during the 2018 comprehensive BRUVS survey; (b) a 3‐factor design including year (two levels; fixed), shoal, and site (nested in shoal) to compare assemblages along the reef slope over time; and (c) a 4‐factor design incorporating year, shoal, site (nested in shoal), and deployment (random; nested in site) to compare assemblages over time in the lagoon. We did not compare fish assemblages in the mesophotic reef habitat through time due to differences in image quality impacting species detection and identification (see Section 2.4).

Multivariate fish species abundance data were fourth‐root transformed to reduce the influence of highly abundant species after data visualization using shade plots. Similarities between species were based on the Bray–Curtis resemblance matrix as it does not treat the absences of species as similarities and emphasizes the composition of the assemblage rather than the relative abundance of individual species (Anderson et al., 2008). Significance levels were obtained using 9,999 permutations of the data for each term with type III sums of squares and permutation of residuals under a reduced model in the PRIMER v7 software with the PERMANOVA add‐on (Anderson et al., 2008; Clarke & Gorley, 2006). Multivariate patterns were further explored by characterization of multivariate data using metric multidimensional scaling (MDS) with vectors representing fish species with greatest influence on observed differences overlaid (Pearson correlations > 0.55 with MDS axes).

Euclidean distance resemblance matrices were constructed with raw or square‐root‐transformed (for heterogeneous data) relative abundances of individual species using PRIMER (Anderson et al., 2008). Temporal patterns at the Rowley Shoals were analyzed with a 4‐factor design with factors year (three levels; fixed), reef habitat, shoal, and site (nested in shoal and reef habitat).

To compare the Rowley Shoals 2018 survey with other remote reefs (five locations), we focused on univariate relative abundance of regionally fished species whose range covered all locations. All selected species are retained for human consumption in both Indonesia and Australia (Rome & Newman, 2010; White et al., 2013). *Cheilinus undulatus* and *Bolbometopon muricatum* were included in surveys at all locations except for Scott Reef where these two species were not included as part of the subset of predatory species recorded for the original project. We used 2‐factor design with location (six levels; fixed) and site (nested in location) to compare locations within each reef habitat. Where multiple pairwise comparisons were conducted (Appendix S4), we used the Benjamini–Hochberg adjustment (Adj. *α*) where the false discovery rate (FDR = 0.05) controls the expected rate of type I error (Benjamini & Hochberg, 1995; Lee & Lee, 2018). Where the number of unique permutations was low, Monte Carlo (MC) *p*‐values were obtained (Anderson et al., 2008).

We compared length distributions among reef habitats at the Rowley Shoals and between the Rowley Shoals and Cocos (Keeling) Islands using kernel density estimates (KDEs) when at least 20 individuals were observed in each level, using the “ggplot2” package in R to estimate the probability density function of the length‐frequency data (Wickham, 2016). Bandwidths were chosen using a “plug‐in” style selection that did not make assumptions about the distributions of the data (Sheather & Jones, 1991). Outliers greater than 1,600 mm (eight sharks) affected the selection of appropriate bandwidths and were therefore removed from analysis (Bond et al., 2018). Bandwidths were then estimated using the “dpik” function in the R package “KernSmooth” (Wand, 2015). We compared the area between the two sets of KDEs using permutations of the data as random pairs. *p*‐values were obtained following the approach outlined by Langlois et al., (2012) using the function “sm.density.compare” in the R package “sm” (Bowman & Azzalini, 2018).

## 3 RESULTS

### 3.1 Rowley Shoals fish assemblages

**FIGURE 2:**
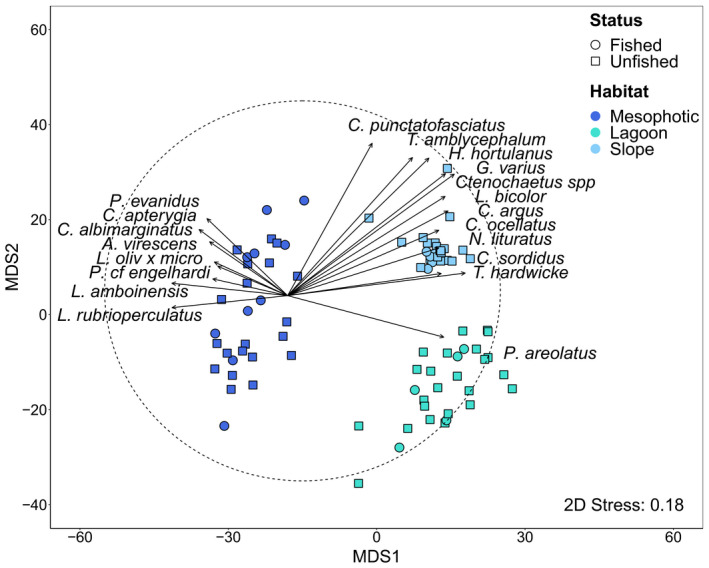
Metric MDS ordination of the Rowley Shoals BRUVs illustrating patterns among reef habitat and management status from 2018 BRUV deployments; species with the strongest positive (> 0.55) and negative (<−0.55) Pearson's correlation values are displayed as vectors

Surveys of the Rowley Shoals in 2018 recorded 14,500 fish from 327 different species. Assemblages were similar among the three shoals (*F*_(2,70)_ = 1.31, *p*_PERM_ = 0.27), and there was no effect of fishing (*F*_(2,70)_ = 1.07, *p*_PERM_ = 0.41; Table 2). However, distinct fish assemblages characterized the different habitats (Table 2; *F*_(2,70)_ = 6.92, *p*_PERM_ < 0.001). Lagoon assemblages were characterized by high abundances of the squaretail coral trout, *Plectropomus areolatus*, while the reef slope supported several abundant herbivorous (e.g., *Cetoscarus ocellatus*, *Ctenochaetus* spp.) and invertivorous species (e.g., *Halichoeres hortulanus*, *Gomphosus varius*). The mesophotic habitat was characterized by several carnivorous (e.g., *Lethrinus amboinensis, Lethrinus rubrioperculatus*) and zooplanktivorous species (e.g., *Pseudanthias cf. engelhardi*, *Conniella apterygia*) (Figure 2). There was no change in the slope and lagoon assemblages between 2013 and 2018 (*F*_(1,49)_ = 3.45, *p*_PERM_ = 0.21 and *F*_(1,19)_ = 1.86, *p*_PERM_ = 0.23, respectively; Table 2). MDS ordinations illustrating patterns between years within both lagoon and slope habitats had high 2D stress values (0.26 and 0.23, respectively) and were therefore not meaningful to present in an ordination.

**TABLE 2:** Results of permutational multivariate analyses of variance examining assemblage composition based on fish abundance data from BRUVS at the Rowley Shoals

	Source	*df*	MS	Pseudo‐*F*	*p*(perm)
4‐factor 2018 BRUVS deployments	Status	1	3,978	1.07	0.41
	Shoal	2	4,887	1.31	0.27
	Habitat	2	26,459	6.92	**<0.001**
	Status × Shoal	1	5,220	1.36	0.29
	Status × Habitat	2	3,447	0.92	0.54
	Shoal × Habitat	4	3,824	1.03	0.47
	Site(Shoal × Habitat × Status)	5	3,772	2.29	**<0.001**
	Res	70	1,643		
	Total	87			
3‐factor 2013 and 2018 slope comparison	Year	1	5,930	3.45	0.21
	Shoal	2	4,021	1.63	0.11
	Site(Shoal)	3	2,237	1.47	**0.02**
	Year × Shoal	2	2,187	1.37	0.40
	Year × Site(Shoal)	1	1,528	1.01	0.43
	Residual	49	1,519		
	Total	58			
4‐factor 2013 and 2018 lagoon comparison	Year	1	4,172.9	1.86	0.23
	Shoal	2	6,003.1	1.49	0.19
	Site(Shoal)	3	4,039.4	2.28	**<0.001**
	Year × Shoal	2	1,963.1	0.87	0.60
	Deployment(Site(Shoal))	24	1,821.6	1.38	**0.001**
	Year × Site(Shoal)	2	2,353.3	1.78	**0.01**
	Residual	19	1,320.6		
	Total	53			

Note

Significant effects are shown in bold.

### 3.2 Rowley Shoals regionally fished species abundance

**FIGURE 3:**
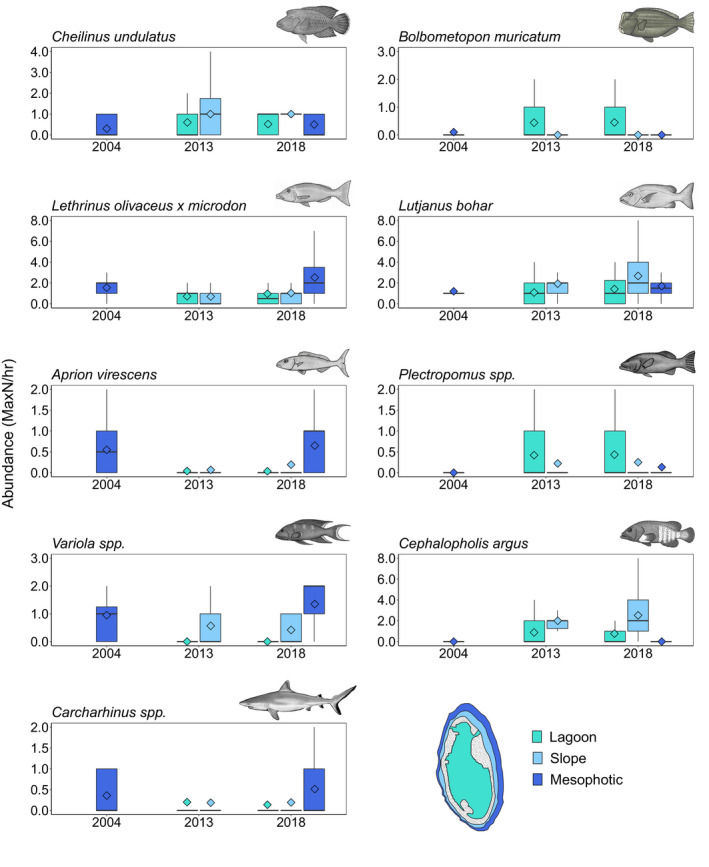
Boxplots with the mean (◊) and median (|) abundance of regionally fished species observed from BRUV surveys at the Rowley Shoals (Pooled across Clerke, Imperieuse and Mermaid shoals) in 2004, 2013, and 2018, in lagoon, reef slope, and mesophotic habitats. Upper and lower hinges represent the first and third quartiles (the 25 and 75 percentiles). The whiskers extend from the hinge to the largest and smallest value, but no further than 1.5× the interquartile range

The relative abundance of selected regionally fished species was consistent between years in all of the reef habitats at the Rowley Shoals, but had marked differences in abundance among habitats (Appendix S3; Figure 3). Shoals were pooled in Figure 3 for ease of interpretation with all species having similar abundances across the shoals except for *C. undulatus* (*F*_(2,133)_ = 5.49, *p*_PERM_ = 0.03) and *Lutjanus bohar* (*F*_(2,133)_ = 5.73, *p*_PERM_ = 0.03) with lower abundances observed at Mermaid and Clerke, respectively. The endangered species *C. undulatus* was present across all habitats with highest abundances on the slope in both 2013 and 2018. The vulnerable species, *B. muricatum,* was present in mesophotic habitat in low numbers in 2004 and in higher abundance in the lagoon in 2013 and 2018. Mesopredators, *Lethrinus olivaceus* × *microdon* (longnose emperor)*, Aprion virescens* (green jobfish), and *Carcharhinus* spp. (requiem sharks) were present in all reef habitats, but most abundant in the mesophotic habitat. *Lutjanus bohar* (red bass) were present at all reef habitats with highest abundances on the reef slope. Similarly, *Plectropomus* spp. (coral trout) were present across all reef habitats but were most common in the lagoon and rare in the mesophotic habitat. *Variola* spp. (coronation trout) were most abundant in the mesophotic habitat and absent from the lagoon. *Cephalopholis argus* (peacock rockcod) were most abundant on the reef slope and were not recorded in mesophotic habitats.

### 3.3 Comparison of regionally fished species among remote locations

**FIGURE 4:**
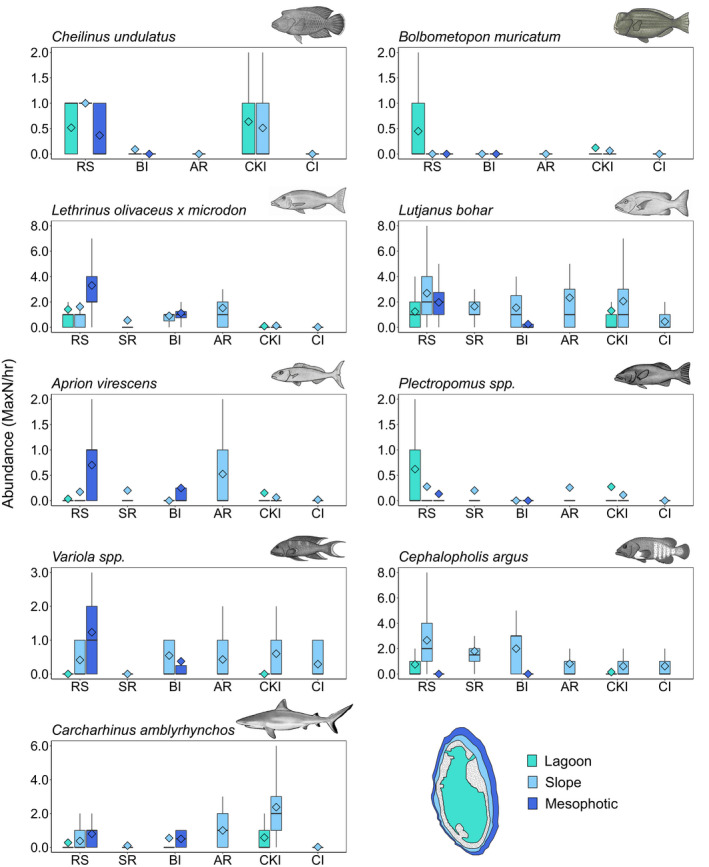
Boxplots with the mean (◊) and median (|) abundance of regionally fished species observed from BRUV surveys at the Rowley Shoals (RS), Scott Reef (SR), Browse Island (BI), Ashmore Reef (AR), Cocos (Keeling) Islands (CKI), and Christmas Island (CI). Upper and lower hinges represent the first and third quartiles (the 25 and 75 percentiles). The whiskers extend from the hinge to the largest and smallest value, but no further than 1.5× the interquartile range

The Rowley Shoals had significantly higher abundances of *C. undulatus* on the reef slope compared with all other locations (*p* < 0.05; Appendix S4), with none observed at Ashmore Reef or Christmas Island. Similar abundances of this species were observed in lagoonal sites at the Rowley Shoals and Cocos (Keeling) Islands (Figure 4). *Cheilinus undulatus* were observed in mesophotic depths at the Rowley Shoals with none observed in the Browse Island mesophotic zone.

*Bolbometopon muricatum* were only observed at the Rowley Shoals and Cocos (Keeling) Islands. No *B. muricatum* were observed on the slope at the Rowley Shoals, and they were rare on the slope at Cocos (Keeling) with only five individuals observed across the 80 deployments.

The Rowley Shoals had significantly higher abundances of *Lethrinus olivaceus* × *microdon* on the reef slope compared with Cocos (Keeling) Islands (*t* = 2.85, *p* = 0.01) and Christmas Island (*t* = 3.68, *p* < 0.001) (Figure 4). At the Rowley Shoals, abundance of *L. olivaceus* × *microdon* was also greater in lagoons than at Cocos (Keeling) and in the mesophotic habitat than at Browse Island, although neither of these differences were statistically significant (*t* = 1.73, *p* = 0.08 and *t* = 2.31, *p*_MC_ = 0.06, respectively; Appendix S4).

Similar abundances of *L. bohar* were observed on the slope at all locations, except for Christmas Island (*t* = 6.71, *p* < 0.001), where abundances were more than six times lower. At the Rowley Shoals, abundance of *L. bohar* in the lagoon was similar to Cocos (Keeling), but was greater in the mesophotic habitat than at Browse Island (*t* = 2.64, *p*_MC_ = 0.05; Appendix S4).

*Aprion virescens* occurred on the slope at all reefs except at Browse Island where they were only in the mesophotic habitat. Abundance of *A. virescens* was particularly high on the reef slope at Ashmore Reef where estimates were three times those at Rowley Shoals and Scott Reef, the only other locations with mean abundance > 0.1 in this habitat. However, abundance of this species in the Rowley Shoals mesophotic zone was similar to that recorded on Ashmore Reef slopes and greater than, but not significantly different (*t* = 1.65, *p*_MC_ = 0.27; Appendix S4), from estimates in the mesophotic habitat at Browse Island. Christmas Island had significantly lower abundances of *A. virescens* than all other locations except for Cocos (Keeling).

Rowley Shoals, Scott Reef, and Ashmore Reef had similar abundances of *Plectropomus* spp. on the slope, with fewer observed at Cocos (Keeling) (Figure 4). Rowley Shoals had a higher, but not significantly different (*t* = 2.04, *p* = 0.07; Appendix S4), abundances of *Plectropomus* spp. in the lagoon than at Cocos (Keeling). *Plectropomus* spp. was not observed at Browse or Christmas Island.

*Variola* spp. were not observed in the lagoon at any location and were absent from Scott Reef. The other locations had similar abundances observed on the slope.

The Rowley Shoals, Scott Reef, and Browse Island had similar abundances of *C. argus* on the reef slope which were higher than at Ashmore Reef, Cocos (Keeling), and Christmas Island.

Cocos (Keeling) had more than twice the abundance of *Carcharhinus amblyrhynchos* compared with all other locations. Grey reef sharks were predominantly on the slope, with abundance in the lagoon at Cocos (Keeling) similar to abundance at the Rowley Shoals. High abundance of *C. amblyrhynchos* was also found in the mesophotic habitat at the Rowley Shoals and Browse Island.

### 3.4 Predator species length distributions among habitats at the Rowley Shoals

**FIGURE 5:**
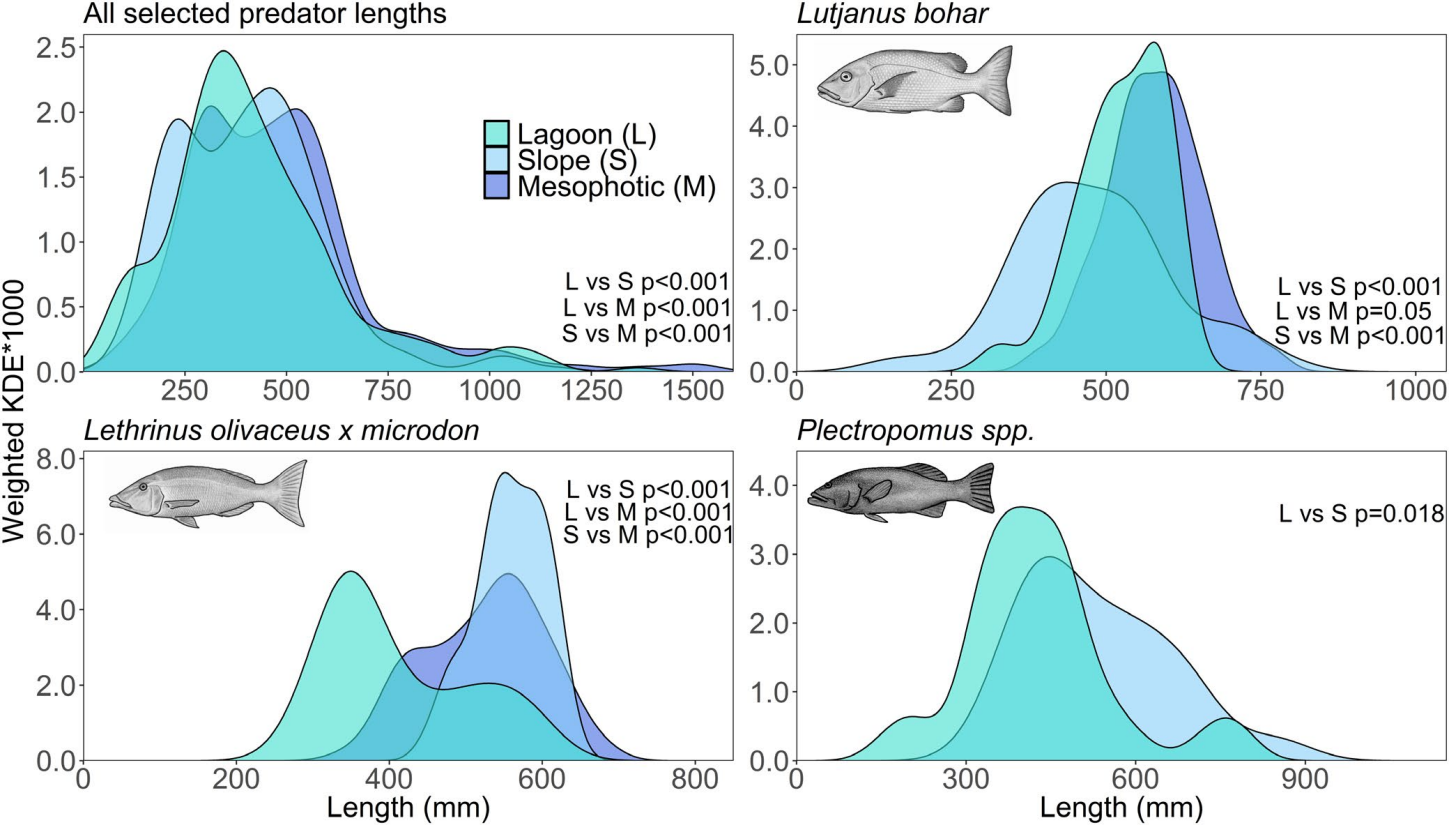
Weighted kernel density estimates (KDE * 1,000) for selected predator fish lengths in three reef habitats at the Rowley Shoals in 2018

Larger predatory fishes were observed in the mesophotic zone with smaller individuals observed in slope and lagoon habitats (Figure 5). Eight large sharks (>1,600 mm) were excluded from this analysis, of which seven were present in the mesophotic habitat. The lagoon had higher density of the smallest (<175 mm) and medium‐sized (250–450 mm) fish. The slope had higher estimates of small (175–250 mm) fish, and the larger (550–750 mm) fish were most abundant in mesophotic samples. Of the three predator taxa that were recorded frequently enough for independent distribution plots, *L. bohar* occurred at a wide range of lengths on the reef slope with a higher proportion of smaller individuals in this habitat than either the lagoon or mesophotic habitats; larger individuals were mostly found in the mesophotic habitat. Conversely, *L. olivaceus* × *microdon* were smaller (<400 mm) in the lagoon than in the slope and mesophotic habitats. *Plectropomus* spp. also had higher density of medium and smaller (<500 mm) fish in the lagoon, and higher density of large (>500 mm) fish on the reef slope.

### 3.5 Predator species length distributions at Rowley Shoals and Cocos (Keeling) Islands

**FIGURE 6:**
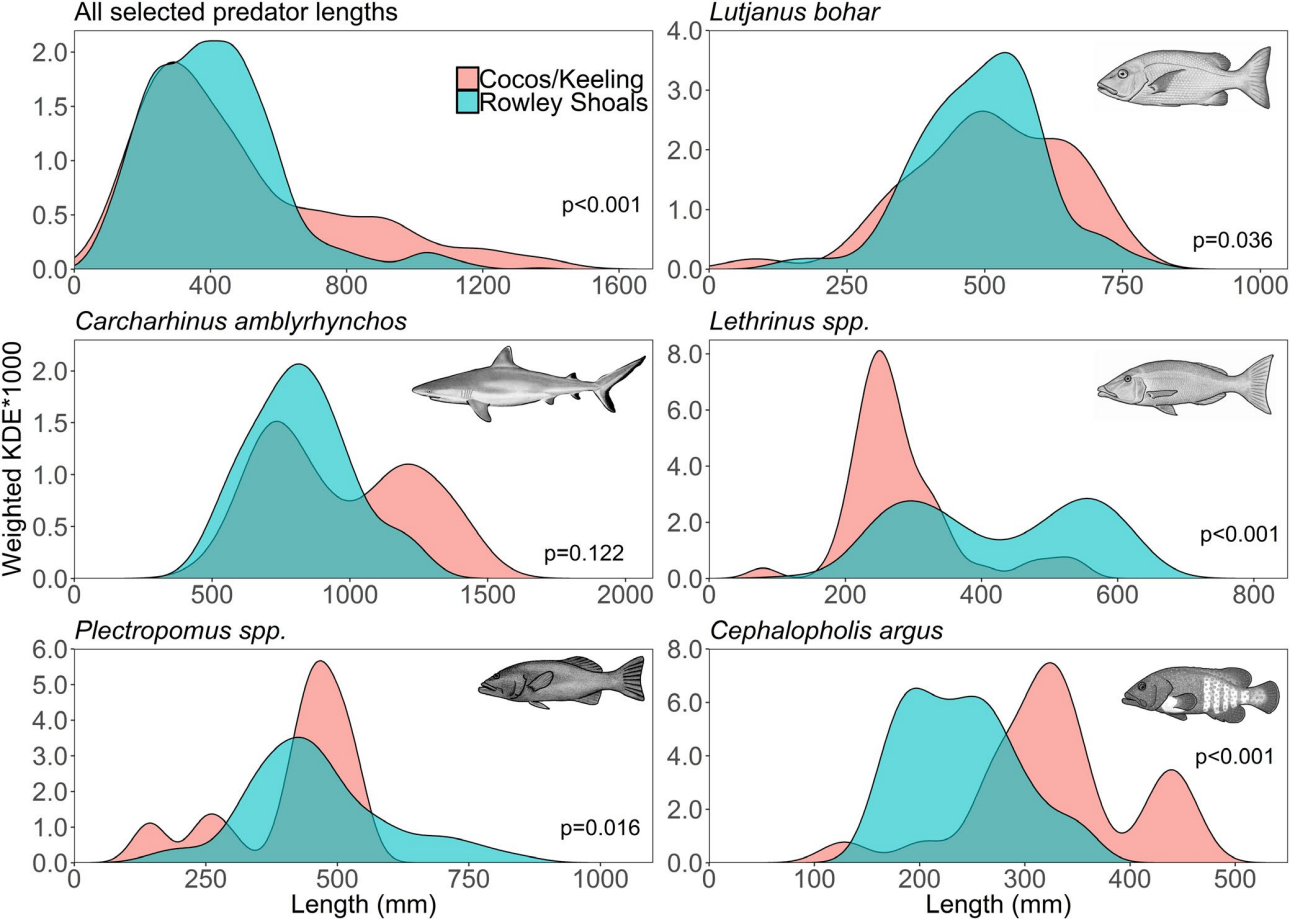
Weighted kernel density estimates (KDE * 1,000) for selected predators observed in the lagoon and on the slope at the Rowley Shoals in 2018 and Cocos (Keeling) Islands in 2016/18

Length distributions for selected predator species differed between Rowley Shoals and Cocos (Keeling) when data for all species recorded on slope and lagoon habitats were combined (Figure 6). A higher proportion of medium‐to‐large (300–700 mm) fish were observed at the Rowley Shoals and a higher proportion of very large (>700 mm) fish at Cocos (Keeling). Density estimates of large *L. bohar* (>600 mm), *C. amblyrhynchos* (>1,100 mm), and *C. argus* (>280 mm) were higher at Cocos (Keeling), whereas the proportion of large *Lethrinus* spp. (>350 mm) and *Plectropomus* spp. (>600 mm) was higher at the Rowley Shoals.

## 4 DISCUSSION

The abundance of large‐bodied and iconic species, many targeted by fishers, has not changed at the Rowley Shoals across three surveys spanning 14 years. This includes the endangered and vulnerable *C. undulatus* and *B. muricatum* (Chan et al., 2012; Russell, 2004). The abundance of these species, as well as most other regionally fished species analyzed, was higher at the Rowley Shoals than at five other remote reef systems in the northeast Indian Ocean. This stability and high abundance of fish populations was evident in each of the distinct lagoon, slope, and mesophotic fish assemblages. This suggests that the Rowley Shoals is a rare example of a reef system that meets all the “NEOLI” (no‐take, enforced, old reserves, large reserves, and isolated) criteria for successful marine conservation (Edgar et al., 2014), which are all contributing to observed temporal stability.

Coral cover has remained consistently high at the Rowley Shoals with an increase of ~20% coral cover during our 14‐year sampling period despite bleaching and cyclonic activity that has caused declines on reef systems at Christmas Island, Ashmore Reef, Scott Reef, and other locations along the WA coast (Gilmour et al., 2019). This is important, as coral communities and their associated structural complexity provide habitat for many coral reef fishes (Pratchett et al., 2008), including fish recruits for which live coral is an essential habitat during early postsettlement life‐history stages (Jones et al., 2004; Wilson et al., 2016). While we found that fish assemblages at the Rowley Shoals remained stable, Ruppert et al., (2013) documented changes in herbivore, corallivore, and planktivore densities following a severe cyclone in 1996 which resulted in lower coral cover (<30%) than was observed during our survey period (>50% cover; Gilmour et al., 2019). Temporal changes in fish assemblages have been documented at other remote locations in the eastern Indian Ocean, such as Ashmore Reef (Speed et al., 2018) and Scott Reef (Halford & Caley, 2009). These community changes are likely due to a combination of reduced fishing pressure at Ashmore Reef and damage to habitats by heat stress and cyclone events at both locations (Gilmour et al., 2019). These changes can effect species with key functional roles, such as herbivores and piscivores (Garpe et al., 2006; Gilmour et al., 2013; Wilson et al., 2019) and persist for extended periods, contributing to long‐lasting shifts in ecosystem function (Robinson et al., 2019).

Lagoons provide a unique environment that supports a range of coral habitats essential for many juvenile and adult fish species. The Rowley Shoals have 92 km^2^ of enclosed lagoon habitat, with a variety of microhabitat shelters to facilitate recruitment of diverse reef fishes. This includes highly valued species such as *P. areolatus* with its juvenile phase relying almost exclusively on coral rubble habitats (Tupper, 2007) as well as *B. muricatum* and *C. undulatus* which recruit into branching corals within wave‐sheltered environments (Bellwood & Choat, 2011; Hamilton et al., 2017; Tupper, 2007). These microhabitats are characteristic of the Rowley Shoals lagoons (Morrison, 2009).

We recorded high abundances of *C. undulatus* and *B. muricatum* at the Rowley Shoals; the Cocos (Keeling) Islands were the only other location with a notable number of these species. Both species are sensitive to fishing pressure, being large‐bodied, slow‐growing, and late‐maturing (Bellwood & Choat, 2011; Fenner, 2014; Hamilton et al., 2019; Sadovy de Mitcheson et al., 2019). The ecological roles of these iconic species contribute to healthy ecosystem function. *Cheilinus undulatus* shapes benthic communities via predation on mollusks, crustaceans, and echinoids, including the crown of thorns starfish (Cowan et al., 2017; Kayal et al., 2012; Kroon et al., 2020; Randall et al., 1978), while *B. muricatum* is the most important bioeroder and the largest coral predator (Bellwood & Choat, 2011), holding a keystone role in the maintenance of coral community structure and stability of coral reefs (Bellwood et al., 2012). Consistent with previous studies that used underwater visual census techniques in shallow water, our study using BRUVS identified high abundances of these large iconic labrids at the Rowley Shoals and relatively low abundances at other atolls and islands (Bellwood et al., 2012; Edgar et al., 2017).

Relative abundances of other regionally fished species were generally high at Rowley Shoals. Given fishing for these species is prohibited at the Rowley Shoals, the high abundance of epinephelids (genera *Plectropomus*, *Variola,* and *Cephalopholis*) indicates that fishing pressure on remote reefs may have an influence on the abundance of these taxa. However, differences in geography and island morphology among these reef systems may also contribute to the differences in abundance across locations (Bennett et al., 2018). For example, the absence of *Plectropomus* spp. at both Browse and Christmas Islands is likely driven by a lack of lagoonal habitats in these locations (Hobbs et al., 2014). However, the presence of lagoon habitat may not always lead to high abundance of target species, particularly in the absence of enforced protection from fishing (e.g., Hamilton et al., 2019). For example, fishing in the Cocos (Keeling) lagoon may be contributing to the lower abundances of *Plectropmous* spp. and *Lethrinus olivaceus* × *microdon*. Understanding the relative importance of fishing and habitat on mesopredators is complex and may be further confounded when the abundance of apex predators is high, as was observed at Cocos (Keeling) Islands (Robbins & Renaud, 2016; Speed et al., 2019).

The reason for the high *C. amblyrhynchos* abundance at Cocos (Keeling) is unclear and an exception to the population status of this species at a global scale (Simpfendorfer & Dulvy, 2017). Previous studies have attributed high abundances of sharks at Cocos (Keeling) to a lack of historical fishing pressure (Robbins et al., 2006). However, in this study, sharks were significantly more abundant at Cocos (Keeling) than at other reefs with long‐term protection in the region. Other factors such as geographical position, oceanography, and island morphology may be contributing to this pattern. Moreover, historically high‐intensity shark fishing in Indonesia and north‐west Australia may have disrupted the connectivity of shark populations with lower‐latitude reefs such as the Rowley Shoals, slowly decreasing the observed populations (Momigliano et al., 2017).

As at many reefs around the world, there are clear differences in fish assemblages and regionally fished species among habitats at the Rowley Shoals. Spatial differences in community structure relate to different environmental conditions and regimes of disturbance. Accordingly, programs tasked with monitoring the effect of stressors on coral reefs typically focus on areas of high ecological value, where impacts are likely to be the greatest (Emslie et al., 2008; Frade et al., 2018; Simpson et al., 2015) while recognizing that this may not be representative of the entire fish assemblage from that reef. This approach may be problematic if species move between habitats or migrate to deeper depths following disturbance. We show that BRUVS are a useful tool for understanding relative abundance and fish lengths across a range of habitats, including the deeper mesophotic zone which is generally not accessible to divers but is an important habitat for many species (Lindfield et al., 2016).

The use of BRUVS in monitoring programs may also broaden our understanding of fish migrations. Lagoons are often thought to be key habitats for juvenile fish, which then move onto the reef slope and into deeper water as they increase in size and age (Skinner et al., 2020). However, ontogenetic shifts among reef habitats have not been fully explored for most fish species and the variation in size classes among habitats at the Rowley Shoals suggests different patterns of recruitment and migration. For example, the smallest *L. bohar* were found on the reef slopes at the Rowley Shoals, with larger individuals found in all three habitats, suggesting recruitment to the slope and migration to multiple different habitats. Indeed, there is evidence to suggest mesophotic reefs act as a nursery for some species which use deepwater black corals as a refuge from predators (Rosa et al., 2016). This may explain the broad size distributions of *Variola* spp. in the mesophotic habitat compared with rare but only large individuals on the reef slope (Appendix S1). Identifying these key habitats for fish at different life‐history stages provides important information for management but requires methods, such as BRUVS, that can provide comparable estimates across all relevant reef habitats and depths.

Our comparison of length frequencies between the Rowley Shoals and Cocos (Keeling) indicates that predatory fish at Cocos (Keeling) are typically larger than conspecifics at the Rowley Shoals. This is especially true of *C. amblyrhynchos*, *L. bohar,* and *C. argus*. Conversely, the largest *Lethrinus* spp. and *Plectropomus* spp. are found at the Rowley Shoals, which may relate to differences in a species phenotypic expression correlated with latitude (Cappo et al., 2013), but is likely influenced by differences in species rather than body size of the same species. Notably, *Plectropomus* spp. and *Lethrinus* spp. are thought to be among the most impacted species at Cocos (Keeling) due to fishing (Department of Fisheries, 2005), and lack of the larger‐bodied species and individuals may be at least partly attributed to fishing pressure. Previous studies have shown the biomass of higher trophic levels to be greater at remote, unfished reefs with warmer waters, and high primary productivity (Friedlander & DeMartini 2002; Heenan et al., 2019; Stevenson et al., 2007). However, the many complex environmental and physical factors that make a reef system will cause differences in the natural state of a reef in the absence of humans and further research will be required to disentangle the key drivers of the differences detected in the predatory fish lengths seen here (Williams et al., 2015).

## 5 CONCLUSION

The Rowley Shoals is a rare reef system that meets the criterion set for global conservation targets (Edgar et al., 2014). This study demonstrates temporal stability in fish assemblages (5 years) and regionally fished species (14 years) in lagoon, slope, and mesophotic habitats. The Rowley Shoals also has high abundances of regionally fished species compared with other isolated reefs in the region, including endangered and vulnerable *C. undulatus* and *B. muricatum*. Lagoon habitats appear to be important for supporting these and other species, especially during the early stages of their lifecycle. However, variability in abundances and length distributions across reef habitats suggest there are varied ontogenetic shifts and habitat preferences among species, some of which include mesophotic habitats. Differences among the locations seen here are likely due to multiple factors such as geomorphology, geographical location, and historical fishing pressure. Importantly, isolation from human populations does not necessarily translate to conditions that support stable fish assemblages and abundances of regionally fished species, further highlighting the rarity of reef systems like the Rowley Shoals and the importance of developing meaningful baselines to quantify the impacts of disturbances on coral reefs.

## CONFLICT OF INTEREST

The authors declare that they have no known competing financial interests or personal relationships that could have appeared to influence the work reported in this paper.

## AUTHOR CONTRIBUTIONS

**Matthew J. Birt:** Conceptualization (lead); data curation (lead); formal analysis (lead); investigation (lead); methodology (lead); project administration (lead); visualization (lead); writing – original draft (lead); writing – review and editing (equal). **Katherine Cure:** Conceptualization (equal); investigation (equal); methodology (equal); visualization (equal); writing – original draft (equal); writing – review and editing (equal). **Shaun Wilson:** Conceptualization (equal); investigation (equal); methodology (equal); validation (equal); visualization (equal); writing – original draft (equal); writing – review and editing (equal). **Stephen J. Newman:** Data curation (equal); investigation (equal); methodology (equal); validation (equal); writing – review and editing (equal). **Euan S. Harvey:** Data curation (equal); funding acquisition (equal); methodology (equal); validation (equal); writing – review and editing (equal). **Mark Meekan:** Data curation (equal); funding acquisition (equal); methodology (equal); validation (equal); writing – review and editing (equal). **Conrad Speed:** Data curation (equal); methodology (equal); validation (equal); writing – review and editing (equal). **Andrew Heyward:** Data curation (equal); funding acquisition (equal); methodology (equal); validation (equal); writing – review and editing (equal). **Jordan Goetze:** Conceptualization (equal); methodology (equal); validation (equal); writing – review and editing (equal). **James Gilmour:** Conceptualization (equal); funding acquisition (equal); investigation (equal); methodology (equal); project administration (equal); resources (equal); validation (equal); writing – review and editing (equal).

## Supporting information

Figure S1

Figure S2

Supplementary Material

## Data Availability

The data used in this study are available at GlobalArchive (https://globalarchive.org/geodata/data/campaign/get/991).
