# Supplementary material for: Isolated reefs support stable fish communities with high abundances of regionally fished species
Source: Ecol Evol. 2021 Mar 16;11(9):4701–18. doi: 10.1002/ece3.7370 (PMC8093692; doi:10.1002/ece3.7370)
Supplement: Supplementary file 3 — Supplementary Material [file ECE3-11-4701-s003.docx]

# Supplementary material





Appendix S1. Length frequency histograms for selected predatory species in three reef habitats at the Rowley Shoals in 2018 (left of centre) and for the slope and lagoon combined at the Rowley Shoals and Cocos (Keeling) Islands (right of centre).





Appendix S2. Boxplots with the mean (◊) and median (|) abundance of regionally fished species at the Cocos (Keeling) Islands at sites with high (S) and moderate-low (NE and NW) levels of wave exposure. Upper and lower hinges represent the first and third quartiles (the 25 and 75 percentiles). The whiskers extend from the hinge to the largest and smallest value, but no further than 1.5 × the interquartile range. Single factor (site; 3 levels) PERMANOVAs revealed all sites were statistically similar with the exception of the *Variola* spp. where pairwise comparisons revealed significantly higher abundances at S than at NW (t = 2.85, *P*=0.02).

Appendix S3. Results of permutational multivariate analyses of variance examining regionally fished species based on fish abundance data from BRUVS at the Rowley Shoals.

|  | **Source** | **df** | **SS** | **MS** | **Pseudo-F** | **P(perm)** |
| --- | --- | --- | --- | --- | --- | --- |
| *Cheilinus undulatus* | Year | 2 | 0.88 | 0.44 | 0.96 | 0.44 |
|  | Shoal | 2 | 3.21 | 1.60 | 5.49 | **0.03** |
|  | Habitat | 2 | 6.49 | 3.24 | 10.93 | **0.01** |
|  | YearxShoal | 3 | 1.02 | 0.34 | 0.75 | 0.56 |
|  | YearxHabitat | 1 | 0.01 | 0.01 | 0.04 | 0.84 |
|  | ShoalxHabitat | 4 | 2.14 | 0.53 | 1.79 | 0.22 |
|  | Site(ShoalxHabitat) | 10 | 3.11 | 0.31 | 1.29 | 0.24 |
|  | YearxShoalxHabitat | 2 | 0.27 | 0.14 | 0.31 | 0.75 |
|  | YearxSite(ShoalxHabitat) | 5 | 2.24 | 0.45 | 1.86 | 0.10 |
|  | Res | 133 | 32.15 | 0.24 |  |  |
|  | Total | 164 | 53.79 |  |  |  |
|  |  |  |  |  |  |  |
| *Bolbometopon muricatum* | Year | 2 | 0.14 | 0.07 | 0.13 | 0.87 |
|  | Shoal | 2 | 1.07 | 0.53 | 1.49 | 0.28 |
|  | Habitat | 2 | 4.30 | 2.15 | 5.69 | **0.03** |
|  | YearxShoal | 3 | 0.27 | 0.09 | 0.17 | 0.91 |
|  | YearxHabitat | 1 | 0.02 | 0.02 | 0.06 | 0.82 |
|  | ShoalxHabitat | 4 | 2.41 | 0.60 | 1.58 | 0.25 |
|  | Site(ShoalxHabitat) | 10 | 4.30 | 0.43 | 2.51 | **0.01** |
|  | YearxShoalxHabitat | 2 | 0.17 | 0.09 | 0.17 | 0.85 |
|  | YearxSite(ShoalxHabitat) | 5 | 2.70 | 0.54 | 3.15 | **0.01** |
|  | Res | 133 | 22.80 | 0.17 |  |  |
|  | Total | 164 | 41.90 |  |  |  |
|  |  |  |  |  |  |  |
| *Lethrinus olivaceus x microdon* | Year | 2 | 1.04 | 0.52 | 0.59 | 0.58 |
|  | Shoal | 2 | 1.20 | 0.60 | 0.69 | 0.55 |
|  | Habitat | 2 | 17.61 | 8.80 | 9.59 | **<0.01** |
|  | YearxShoal | 3 | 1.04 | 0.35 | 0.40 | 0.75 |
|  | YearxHabitat | 1 | 0.09 | 0.09 | 0.12 | 0.75 |
|  | ShoalxHabitat | 4 | 12.44 | 3.11 | 3.36 | 0.07 |
|  | Site(ShoalxHabitat) | 10 | 10.47 | 1.05 | 2.57 | **0.01** |
|  | YearxShoalxHabitat | 2 | 1.29 | 0.64 | 0.73 | 0.52 |
|  | YearxSite(ShoalxHabitat) | 5 | 4.34 | 0.87 | 2.13 | 0.07 |
|  | Res | 133 | 54.29 | 0.41 |  |  |
|  | Total | 164 | 125.16 |  |  |  |
|  |  |  |  |  |  |  |
| *Lutjanus bohar* | Year | 2 | 2.47 | 1.23 | 0.66 | 0.55 |
|  | Shoal | 2 | 29.17 | 14.59 | 5.73 | **0.03** |
|  | Habitat | 2 | 34.76 | 17.38 | 6.86 | **0.02** |
|  | YearxShoal | 3 | 2.16 | 0.72 | 0.41 | 0.76 |
|  | YearxHabitat | 1 | 0.43 | 0.43 | 0.28 | 0.63 |
|  | ShoalxHabitat | 4 | 41.50 | 10.38 | 4.10 | **0.04** |
|  | Site(ShoalxHabitat) | 10 | 25.06 | 2.51 | 0.95 | 0.49 |
|  | YearxShoalxHabitat | 2 | 0.27 | 0.14 | 0.14 | 0.87 |
|  | YearxSite(ShoalxHabitat) | 5 | 9.70 | 1.94 | 0.73 | 0.60 |
|  | Res | 133 | 352.15 | 2.65 |  |  |
|  | Total | 164 | 525.25 |  |  |  |
|  |  |  |  |  |  |  |
| *Aprion virescens* | Year | 2 | 0.11 | 0.06 | 0.34 | 0.74 |
|  | Shoal | 2 | 0.01 | 0.00 | 0.02 | 0.98 |
|  | Habitat | 2 | 7.55 | 3.77 | 18.18 | **<0.001** |
|  | YearxShoal | 3 | 0.91 | 0.30 | 1.76 | 0.27 |
|  | YearxHabitat | 1 | 0.01 | 0.01 | 0.09 | 0.78 |
|  | ShoalxHabitat | 4 | 2.64 | 0.66 | 3.17 | 0.07 |
|  | Site(ShoalxHabitat) | 10 | 2.20 | 0.22 | 1.37 | 0.20 |
|  | YearxShoalxHabitat | 2 | 0.01 | 0.01 | 0.09 | 0.90 |
|  | YearxSite(ShoalxHabitat) | 5 | 0.85 | 0.17 | 1.06 | 0.39 |
|  | Res | 133 | 21.30 | 0.16 |  |  |
|  | Total | 164 | 39.31 |  |  |  |
|  |  |  |  |  |  |  |
| *Plectropomus spp.* | Year | 2 | 2.40 | 1.20 | 3.49 | 0.11 |
|  | Shoal | 2 | 3.47 | 1.74 | 3.42 | 0.06 |
|  | Habitat | 2 | 16.97 | 8.49 | 15.97 | **<0.01** |
|  | YearxShoal | 3 | 0.40 | 0.13 | 0.41 | 0.76 |
|  | YearxHabitat | 1 | <0.01 | <0.01 | 0.05 | 0.83 |
|  | ShoalxHabitat | 4 | 2.30 | 0.58 | 1.07 | 0.41 |
|  | Site(ShoalxHabitat) | 10 | 5.99 | 0.60 | 2.28 | **0.02** |
|  | YearxShoalxHabitat | 2 | 0.56 | 0.28 | 0.83 | 0.48 |
|  | YearxSite(ShoalxHabitat) | 5 | 1.68 | 0.34 | 1.28 | 0.28 |
|  | Res | 133 | 35.01 | 0.26 |  |  |
|  | Total | 164 | 86.61 |  |  |  |
|  |  |  |  |  |  |  |
| *Variola spp.* | Year | 2 | 1.55 | 0.77 | 2.17 | 0.20 |
|  | Shoal | 2 | 3.30 | 1.65 | 2.46 | 0.11 |
|  | Habitat | 2 | 24.99 | 12.50 | 17.63 | **<0.001** |
|  | YearxShoal | 3 | 1.00 | 0.33 | 0.94 | 0.50 |
|  | YearxHabitat | 1 | 0.01 | 0.01 | 0.06 | 0.82 |
|  | ShoalxHabitat | 4 | 1.80 | 0.45 | 0.63 | 0.68 |
|  | Site(ShoalxHabitat) | 10 | 8.20 | 0.82 | 3.02 | **<0.01** |
|  | YearxShoalxHabitat | 2 | 0.10 | 0.05 | 0.17 | 0.85 |
|  | YearxSite(ShoalxHabitat) | 5 | 1.75 | 0.35 | 1.29 | 0.27 |
|  | Res | 133 | 36.10 | 0.27 |  |  |
|  | Total | 164 | 93.18 |  |  |  |
|  |  |  |  |  |  |  |
| *Cephalopholis argus* | Year | 2 | 0.18 | 0.09 | 0.37 | 0.71 |
|  | Shoal | 2 | 0.42 | 0.21 | 0.38 | 0.70 |
|  | Habitat | 2 | 32.35 | 16.17 | 27.96 | **<0.001** |
|  | YearxShoal | 3 | 0.38 | 0.13 | 0.51 | 0.69 |
|  | YearxHabitat | 1 | 0.04 | 0.04 | 0.21 | 0.67 |
|  | ShoalxHabitat | 4 | 3.82 | 0.96 | 1.64 | 0.25 |
|  | Site(ShoalxHabitat) | 10 | 6.56 | 0.66 | 2.41 | **0.01** |
|  | YearxShoalxHabitat | 2 | 0.24 | 0.12 | 0.49 | 0.65 |
|  | YearxSite(ShoalxHabitat) | 5 | 1.30 | 0.26 | 0.96 | 0.43 |
|  | Res | 133 | 36.14 | 0.27 |  |  |
|  | Total | 164 | 102.11 |  |  |  |
|  |  |  |  |  |  |  |
| *Carcharhinus amblyrhynchos* | Year | 2 | 0.54 | 0.27 | 4.12 | 0.09 |
|  | Shoal | 2 | 1.80 | 0.90 | 2.30 | 0.16 |
|  | Habitat | 2 | 10.69 | 5.34 | 13.32 | **<0.01** |
|  | YearxShoal | 3 | 1.39 | 0.46 | 6.93 | **0.03** |
|  | YearxHabitat | 1 | 0.26 | 0.26 | 3.90 | 0.10 |
|  | ShoalxHabitat | 4 | 0.62 | 0.16 | 0.39 | 0.81 |
|  | Site(ShoalxHabitat) | 10 | 4.29 | 0.43 | 1.47 | 0.16 |
|  | YearxShoalxHabitat | 2 | 1.03 | 0.51 | 7.61 | **0.03** |
|  | YearxSite(ShoalxHabitat) | 5 | 0.33 | 0.07 | 0.23 | 0.95 |
|  | Res | 133 | 38.76 | 0.29 |  |  |
|  | Total | 164 | 62.09 |  |  |  |

Appendix S4. Results of pairwise comparisons examining regionally fished species based on BRUV data from the Rowley Shoals, Browse Island, Scott Reef, Ashmore Reef, Cocos (Keeling) Islands and Christmas Island.

| Groups | **Lagoon** | | **Slope** | | | **Deep** | |
| --- | --- | --- | --- | --- | --- | --- | --- |
| *Cheilinus undulatus* | t | P(perm) | t | P(perm) | Adj. α | t | P(MC) |
| Rowley Shoals, Browse Island |  |  | 4.49 | 0.07 | 0.05 |  |  |
| Rowley Shoals, Scott Reef |  |  | 7.70 | **0.02** | 0.03 |  |  |
| Rowley Shoals, Ashmore |  |  | 9.41 | **0.01** | 0.02 |  |  |
| Rowley Shoals, Cocos/Keeling Islands | 1.10 | 0.38 | 3.13 | **0.03** | 0.04 |  |  |
| Rowley Shoals, Christmas Island |  |  | 17.01 | **<0.001** | 0.01 |  |  |
|  |  |  |  |  |  |  |  |
| *Bolbometopon muricatum* |  |  |  |  |  |  |  |
| Rowley Shoals, Browse Island |  |  |  |  |  |  |  |
| Rowley Shoals, Scott Reef |  |  |  |  |  |  |  |
| Rowley Shoals, Ashmore |  |  |  |  |  |  |  |
| Rowley Shoals, Cocos/Keeling Islands | 1.10 | 0.38 |  |  |  |  |  |
| Rowley Shoals, Christmas Island |  |  |  |  |  |  |  |
|  |  |  |  |  |  |  |  |
| *Lethrinus olivaceus x microdon* |  |  |  |  |  |  |  |
| Rowley Shoals, Browse Island |  |  | 0.32 | 0.62 | 0.04 | 2.31 | 0.06 |
| Rowley Shoals, Scott Reef |  |  | 1.14 | 0.26 | 0.03 |  |  |
| Rowley Shoals, Ashmore |  |  | 0.27 | 0.78 | 0.05 |  |  |
| Rowley Shoals, Cocos/Keeling Islands | 1.73 | 0.08 | 2.85 | **0.01** | 0.02 |  |  |
| Rowley Shoals, Christmas Island |  |  | 3.68 | **<0.001** | 0.01 |  |  |
|  |  |  |  |  |  |  |  |
| *Lutjanus bohar* |  |  |  |  |  |  |  |
| Rowley Shoals, Browse Island |  |  | 1.41 | 0.27 | 0.04 | 2.64 | **0.05** |
| Rowley Shoals, Scott Reef |  |  | 1.01 | 0.31 | 0.05 |  |  |
| Rowley Shoals, Ashmore |  |  | 1.43 | 0.18 | 0.03 |  |  |
| Rowley Shoals, Cocos/Keeling Islands | 0.57 | 0.59 | 1.82 | 0.11 | 0.02 |  |  |
| Rowley Shoals, Christmas Island |  |  | 6.71 | **<0.001** | 0.01 |  |  |
|  |  |  |  |  |  |  |  |
| *Aprion virescens* |  |  |  |  |  |  |  |
| Rowley Shoals, Browse Island |  |  | 1.06 | 0.26 | 0.04 | 1.26 | 0.27 |
| Rowley Shoals, Scott Reef |  |  | 0.57 | 0.66 | 0.05 |  |  |
| Rowley Shoals, Ashmore |  |  | 2.28 | 0.09 | 0.02 |  |  |
| Rowley Shoals, Cocos/Keeling Islands | 1.02 | 0.41 | 1.69 | 0.17 | 0.03 |  |  |
| Rowley Shoals, Christmas Island |  |  | 2.69 | **0.01** | 0.01 |  |  |
|  |  |  |  |  |  |  |  |
| *Plectropomus spp.* |  |  |  |  |  |  |  |
| Rowley Shoals, Browse Island |  |  | 1.08 | 0.16 | 0.02 | 1.23 | 0.28 |
| Rowley Shoals, Scott Reef |  |  | 1.04 | 0.30 | 0.03 |  |  |
| Rowley Shoals, Ashmore |  |  | 0.86 | 0.48 | 0.04 |  |  |
| Rowley Shoals, Cocos/Keeling Islands | 2.04 | 0.07 | 0.82 | 0.49 | 0.05 |  |  |
| Rowley Shoals, Christmas Island |  |  | 3.72 | **0.001** | 0.01 |  |  |
|  |  |  |  |  |  |  |  |
| *Variola spp.* |  |  |  |  |  |  |  |
| Rowley Shoals, Browse Island |  |  | 0.22 | 0.81 | 0.04 | 1.65 | 0.16 |
| Rowley Shoals, Scott Reef |  |  | 1.19 | 0.28 | 0.01 |  |  |
| Rowley Shoals, Ashmore |  |  | 0.52 | 0.44 | 0.03 |  |  |
| Rowley Shoals, Cocos/Keeling Islands |  |  | 0.22 | 0.84 | 0.05 |  |  |
| Rowley Shoals, Christmas Island |  |  | 0.96 | 0.35 | 0.02 |  |  |
|  |  |  |  |  |  |  |  |
| *Cephalopholis argus* |  |  |  |  |  |  |  |
| Rowley Shoals, Browse Island |  |  | 0.63 | 0.39 | 0.04 |  |  |
| Rowley Shoals, Scott Reef |  |  | 0.44 | 0.62 | 0.05 |  |  |
| Rowley Shoals, Ashmore |  |  | 2.70 | 0.04 | 0.03 |  |  |
| Rowley Shoals, Cocos/Keeling Islands | 1.59 | 0.16 | 3.66 | **0.01** | 0.02 |  |  |
| Rowley Shoals, Christmas Island |  |  | 3.74 | **<0.01** | 0.01 |  |  |
|  |  |  |  |  |  |  |  |
| *Carcharhinus amblyrhynchos* |  |  |  |  |  |  |  |
| Rowley Shoals, Browse Island |  |  | 0.99 | 0.27 | 0.05 | 1.15 | 0.38 |
| Rowley Shoals, Scott Reef |  |  | 1.84 | 0.13 | 0.04 |  |  |
| Rowley Shoals, Ashmore |  |  | 2.14 | 0.08 | 0.03 |  |  |
| Rowley Shoals, Cocos/Keeling Islands | 1.25 | 0.26 | 5.07 | **<0.01** | 0.02 |  |  |
| Rowley Shoals, Christmas Island |  |  | 5.15 | **0.001** | 0.01 |  |  |
